# Investigation of storage response of a plant latex based soft candy colored with blue pigment obtained from *Arthrospira (Spirulina) platensis*

**DOI:** 10.1007/s13197-025-06338-4

**Published:** 2025-05-30

**Authors:** Recep Gunes, Ibrahim Palabiyik, Sefik Kurultay, Bayram Yurt

**Affiliations:** 1 Faculty of Engineering, Department of Food Engineering, Kirklareli University, Kirklareli, 39100 Türkiye; 2 Faculty of Agriculture, Department of Food Engineering, Tekirdag Namik Kemal University, Tekirdag, 59030 Türkiye; 3 Faculty of Engineering and Architecture, Department of Food Engineering, Bingol University, Bingol, 12000 Türkiye

**Keywords:** Kenger gum, Toffee production, Phycocyanin, Color stability

## Abstract

In this study, sugared and sugar-free toffee type soft candy samples were produced by means of using a plant-based latex obtained from *Gundelia tournefortii* L. (known as kenger gum) with a new process. As a natural blue color source, different amounts of phycocyanin (0.1, 0.3, and 0.5 g/100 g) were added to candy samples, and various quality parameters were examined by comparing with the conventional toffee sample. According to the results, water activity of all samples belonging to control, kenger gum-added sugared (KS) and sugar-free (KSF) groups were found as 0.5745–0.5957, 0.6297–0.6388, 0.5471–0.5570, respectively. There were significant differences between the L*, a*, and b* values of phycocyanin-free control, KS, and KSF samples. However, phycocyanin was more stable especially in KS group (ΔE, 1.46–2.71) during the storage compared to other two groups. Textural parameters of the samples changed depending on difference in the compositions i.e., kenger gum and sugar alcohols, regardless of phycocyanin concentration. High consumption appeal was achieved by the integration of kenger gum and phycocyanin in toffee type soft candy production. According to the outputs, kenger gum can be effectively employed as a base ingredient in such products and phycocyanin can serve as a natural blue color in the production.

**Graphical Abstract:**

## Highlights

- A special plant latex (kenger gum) was used in toffee type candy production with a new process.
- Phycocyanin was added to the candy samples as a natural blue color source.
- Phycocyanin was found to be quite stable in novel samples produced with plant latex.
- The use of phycocyanin in toffee type soft candy production resulted in high consumption appeal.

## Graphical abstract

**Figure:**
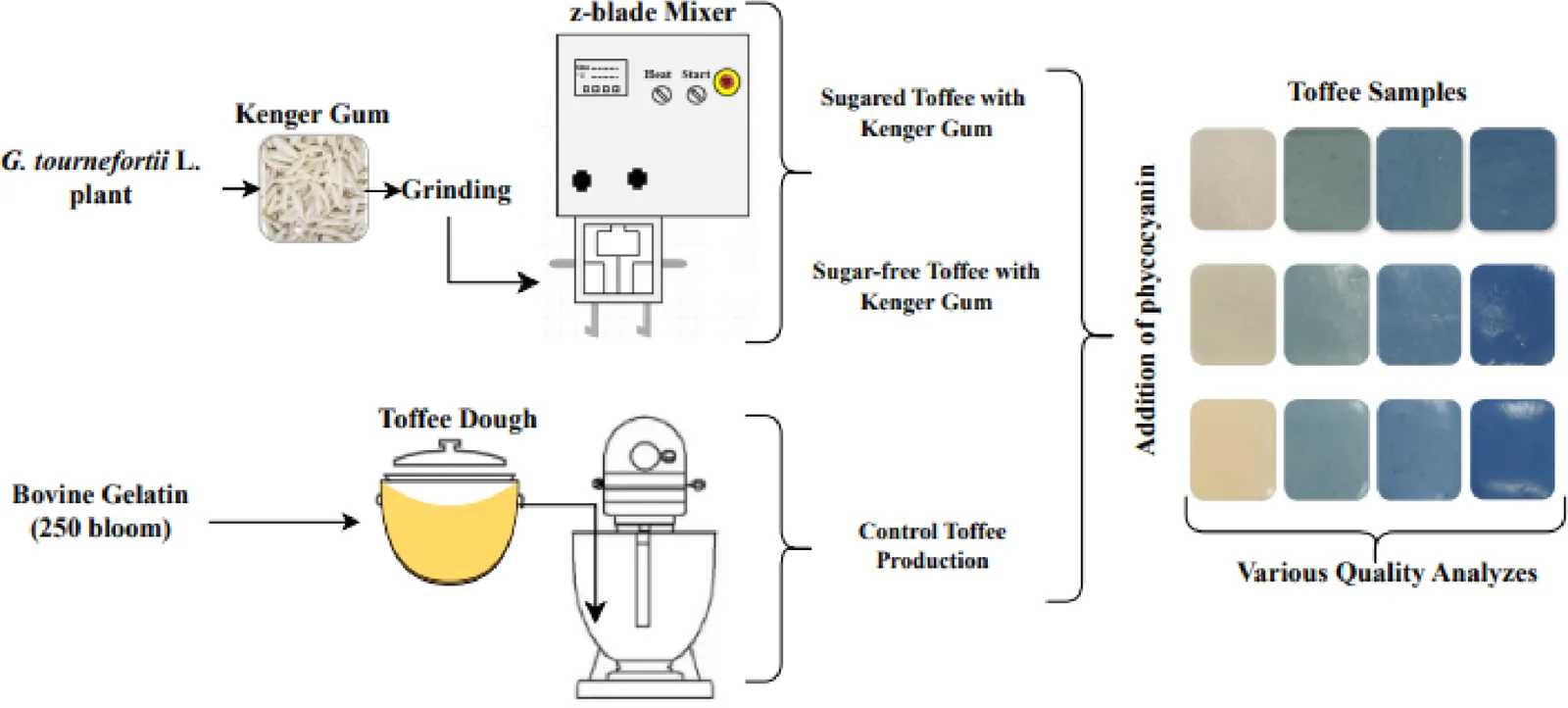

## Introduction

Sugar-based confectionery products encompass a wide variety of high-calorie foods that are chart-topping among people of all ages. Soft candy products, in particular, can be categorized into various sub-groups depending on the ingredients, manufacturing process, texture, and distinctive level of chewiness. This sub-grouping typically includes a diverse range of aerated confections, from lightly aerated candies like toffee/taffy to more heavily aerated marshmallow-type products (Gunes et al. 2022; Hartel et al. 2018). Gelatin is commonly used in these products as a stabilizer to achieve a gummy and elastic texture. However, alternative approaches are often considered due to religious and dietary preferences (such as vegan and vegetarian), potential allergic reactions, and health concerns like Bovine Spongiform Encephalopathy (BSE) (Nhari et al. 2012). As a result, recent years have seen numerous studies focused on finding alternatives to gelatin in these products, leading to the development of innovative production techniques.

In this context, it is seen that the use of a wide variety of extracts, polysaccharides and gum-like materials obtained from different plants is commonly evaluated. Within this perspective, *Gundelia tournefortii* L., a thorny, flowering plant from the Asteraceae family, is recognized for its medicinal properties and is native to the temperate regions of Western Asia. This unique plant, is also known by various names like kangar, kenger, kengel, and kengir. It has versatile uses in the regions where it grows, for instance, its stems, roots, leaves, and young buds can be consumed in various ways and even serve as animal feed. During the summer months (especially July and August), the aboveground parts of the plant dry out and when the part of the plant close to the root is cut during this period, it exudes a viscous white liquid. This substance, when left to solidify, forms a material similar to chewing gum base and is utilized for various purposes by local communities (Çoruh et al. 2007; Evin 2012). Kenger gum is characterized as a plant-based latex composed of high molecular weight polymers structured by the bonding of isoprene units. This organic polymer is insoluble in water, ethyl or methyl alcohol, and acetone. As a result, the structure of kenger gum is primarily understood through empirical methods, particularly using elemental analysis with the FTIR technique. In this regard, it has been determined that kenger gum consists of 60.1% carbon, 8.8% hydrogen, and 30.1% oxygen (Cakmakci and Dagdemir 2013). However, scientific research on the application of kenger gum in food production remains quite limited (Palabiyik et al. 2018). 

Today, the use of synthetic blue dyes in the food industry is restricted and the interest in the use of its natural counterpart is increasing day by day (Jespersen et al. 2005). In this regard, several studies have focused on using *Arthrospira (Spirulina) platensis* directly in powder form or the blue pigment (phycocyanin) that can be extracted from it as a natural food coloring. However, phycocyanin shows low stability to temperature (Mysliwa-Kurdziel and Solymosi 2017) and it is stated that critical temperature for the pigment is 47–50 °C (Jespersen et al. 2005). In the production of toffee type candy, colorants are added at high temperatures after the sugar syrup is cooked. For this reason, since high temperatures will negatively affect phycocyanin in the conventional production, it is necessary to carry out this stage at lower temperatures with an innovative approach or to develop new production techniques. In line with all these, in the present study, production of sugared and sugar-free toffee type soft candy with a new process using kenger gum and a laboratory type mixer (z-blade kneading machine) was performed. In addition, the use of phycocyanin as a natural blue colorant was tried for the first time in these novel samples and then, various quality parameters of the samples were examined by comparing with the conventional control sample during 5 months storage period at 20 ± 2 °C.

## Materials and methods

### Materials

The plant latex (kenger gum) was obtained from local marketers in Malatya, located in the Eastern Anatolia region of Türkiye. Phycocyanin powder used as a natural blue colorant in candy samples was purchased from Meilleur du Chef (Türkiye). A temperature-controlled laboratory size z-blade mixer was developed by Tulpar Machinery (Istanbul, Türkiye) for use in the novel candy production method. Additional ingredients like 42 DE glucose syrup, sorbitol (powder), xylitol (powder), and isomalt (powder) used in the formulations were purchased from Smart Chemistry (İzmir, Türkiye).

### Methods

#### Production of conventional and Kenger gum-added toffee type soft candy samples

The control group formulation includes the following ingredients; 15.10 g of water, 42.70 g of sugar, 3.10 g of sorbitol, 0.8 g of citric acid anhydrate, 31.50 g of glucose syrup, 0.045 g of glycerol monostearate, 0.035 g of soy lecithin, 5 g of hydrogenated vegetable oil, 1.5 g of bovine gelatin (Type A, 250 Bloom), and 0.02 g of salt per 100 g of sample. First, water, sugar, and glucose syrup (42 DE) cooked to 100 °C in a Thermomix (TM5, UK) at 200 rpm. Then, the temperature was increased to 120 °C, and hydrogenated vegetable oil, lecithin, sorbitol, salt, and gelatin were added. Heating and stirring at 200 rpm were kept until the soluble solid concentration reached 90.0 ± 2.00 °Brix. The mixture was then cooled to 90 °C before adding citric acid anhydrate. Subsequently, the mass temperature was decreased to around 50 °C, and 0.20 g/100 g of liquid blueberry flavor was added. Thereafter, the candy dough was rolled and kneaded for 6–8 min using KitchenAid (USA) equipment. Upon completion, the mass was poured into a flat silicon mold, allowed to cool at room temperature, and then cut into equal pieces (1.5 cm × 1.5 cm × 0.5 cm, 2.5 g) (Hartel et al. 2018).

In novel samples containing kenger gum, two different productions (sugared and sugar-free) were conducted. The amounts of kenger gum and other components used in the production of these samples were explained in the previous publication of the authors (Gunes et al. 2024), and a similar path was followed in the current study. Briefly, the composition of kenger gum-added sugared sample (KS) included 2.16 g of kenger gum, 46.72 g of powder sugar, 48.92 g of glucose syrup, 2 g of squalene and 0.2 g of blueberry aroma as per 100 g of sample. On the other hand, the final composition of kenger gum-added sugar-free sample (KSF) consisted of 2 g of kenger gum, 55.81 g of isomalt, 15.00 g of powder sorbitol, 19.99 g of maltitol syrup, 5 g of xylitol, 2 g of squalene, and 0.2 g of blueberry aroma as per 100 g of sample. Considering the production step, the components of the novel samples (100 g for each batch) were weighed and transferred to the chamber of a z-blade mixer. After that, the cooking process was applied at 120 ºC for 40 min. Squalene was added to the mixture starting in the 5th min of cooking. At the end of the cooking process, the temperature of the mixture was reduced to 70 ºC while stirring continued. Meanwhile, thanks to the stirring provided by the z-blade mixer, the pulling step was also carried out on the cooled candy dough. As a result of this process, the pre-cooking of kenger gum with other ingredients was completed and the candy dough was taken from the mixer (Gunes et al. 2024).

### Addition of phycocyanin to the candy products

Considering the amount of coloring additives in soft confectionery products, powder phycocyanin (E18, moisture < 8%, pH = 6) was added to the control sample at the rates of 0.1, 0.2, 0.3, 0.5, and 0.8 g/100 g (w/w) in the pulling process (approximately 50 °C) for the preliminary trial purposes. As a result of this preliminary trial, the rates of 0.1, 0.3, and 0.5 g/100 g remained constant. Unlike the control group, phycocyanin was added to the candy dough of kenger gum-added samples (100 g) at 40 °C, thanks to the advantage of the novel process carried out with the z-blade mixer, and mixing process was applied in the mixer for 1 min. Then, all sample doughs were kept at room temperature for approximately 3–4 h and cut equally (1.5 cm × 1.5 cm × 0.5 cm, 2.5 g). The samples were first wrapped in non-stick food wrapping paper and then metallic-coated polyethylene wrappers. Finally, the samples were packed in plastic bags (polyethylene, PE, 200 G/50 µ) and stored at 20 ± 2 °C for 5 months.

### Water activity (a_w_) analysis

The water activity values of all samples were determined using an a_w_ meter (Labswift, Novasina, Switzerland) immediately after production (1st), 90th and 150th days (Samsiah et al. 2009).

### Color analysis

The color properties of all samples were determined using a color measuring device (Konica Minolta, Bench-top Colorimeter CR-5, Tokyo, Japan). The L* (brightness), a* (± red-green) and b* (± yellow-blue) values were recorded for at least 3 samples in the analysis. Then, ΔE values were calculated by using these obtained values according to Eq. (1) (Pathare et al. 2013).

ΔE = [(ΔL*)^2^ + (Δa*)^2^ + (Δb*)^2^]^1/2^            (1)

### Texture analysis

The instrumental textural characteristics of all samples were analyzed using a Texture Analyzer (TA.HDPlus, Stable Micro Systems, Godalming, Surrey, UK) with P/2E probe (2 mm Dia Cylinder Stainless Extended, 2 mm diameter and 3.14 mm^2^ contact area). First, samples were taken into 3.5 mL containers (1.5 cm diameter and 1.5 cm length) and tested with a 50 kg load cell, 5 mm/s test speed of, 5 g (0.05 N) trigger force, 3 mm depth, and 5 s delay between the first and second penetration. Then, the total work applied was recorded using the force-time curves, and hardness (N), chewiness (N), cohesiveness, adhesiveness (N.sec) values of the samples were determined. The texture analysis was repeated on immediately after production (1st), 90th, and 150th days (Altınok et al. 2020).

### Sensory analysis

In the sensory analysis, the color, taste, hardness, stickiness, and general acceptability characteristics of the samples were examined by 40 experienced panelists (20 females and 20 males, aged 22–36 years) using a 9-point hedonic scale (Shah et al. 2010). For the color, taste, and general acceptability parameters the scale was 1 = dislike extremely, 9 = like extremely; for the hardness parameter it was 1 = extremely soft, 9 = extremely hard; and for the stickiness parameter it was 1 = not sticky, 9 = extremely sticky. During the sensory analysis, samples were categorized using a randomly chosen number, and water and crackers were provided in between each evaluation. The study was authorized by Tekirdag Namik Kemal University, and each panelist provided informed consent before taking part in the study.

### Statistical analysis

The data obtained from the analysis studies were used to determine whether there was a statistical difference between the groups using the Windows-based SPSS 17.0.1 (SPSS Inc., Chicago, Illinois, US) and one-way ANOVA Tukey test (*p* < 0.05).

## Results and discussion

### Water activity results

The water activity (a_w_) values of the samples are given in Table 1 during the storage period. Considering the results, the highest a_w_ values were recorded in the samples belonging to the KS group, followed by the samples in the control group and the samples in the KSF group, respectively (*p* < 0.05). Except for the KSF group, the values of all samples in the control and KS groups decreased from the first day to the end of the storage period, indicating that the samples lost moisture during storage. Similarly, in the study conducted by Kaur et al. (2023), it was stated that the slight decrease in water activity in pumpkin-based toffees produced with non-refined sugar obtained from sugar cane may be due to moisture loss at ambient temperature. On the other hand, in the KSF group, after the 90th day, all samples gained moisture and an increase in a_w_ values was observed (Table 1).

**Table 1:** Water activity values of soft candy samples

Sample	a_w_ value (25 °C)		
	1st day	90th day	150th day
C PC 0	0.5957 ± 0.0013^Da1^	0.5361 ± 0.0013^Db1^	0.4824 ± 0.0011^Dc1^
C PC 0.1	0.5812 ± 0.0016^Fa3^	0.5278 ± 0.0009^Eb2^	0.4744 ± 0.0008^Fc2^
C PC 0.3	0.5745 ± 0.0010^Ga4^	0.5155 ± 0.0009^Fb4^	0.4693 ± 0.0018^Gc3^
C PC 0.5	0.5886 ± 0.0020^Ea2^	0.5242 ± 0.0014^Eb3^	0.4788 ± 0.0010^Ec1^
KS PC 0	0.6388 ± 0.0019^Aa1^	0.5595 ± 0.0014^Ab1^	0.4959 ± 0.0015^Bc1^
KS PC 0.1	0.6314 ± 0.0015^Ca2^	0.5431 ± 0.0011^Cb3^	0.4907 ± 0.0012^Cc2^
KS PC 0.3	0.6297 ± 0.0010^Ca3^	0.5524 ± 0.0013^Bb2^	0.4977 ± 0.0014^ABc1^
KS PC 0.5	0.6345 ± 0.0013^Ba3^	0.5558 ± 0.0016^ABb12^	0.4992 ± 0.0011^Ac1^
KSF PC 0	0.5517 ± 0.0016^Ia2^	0.4151 ± 0.0018^Gc1^	0.4593 ± 0.0007^Hb1^
KSF PC 0.1	0.5471 ± 0.0012^Ja3^	0.4030 ± 0.0020^Ic3^	0.4411 ± 0.0002^Jb3^
KSF PC 0.3	0.5570 ± 0.0014^Ha1^	0.4104 ± 0.0024^GHc2^	0.4445 ± 0.0010^Ib2^
KSF PC 0.5	0.5523 ± 0.0017^Ia2^	0.4063 ± 0.0005^HIc23^	0.4358 ± 0.0009^Kb4^

Data represent average values ± standard deviation of three independent samples

^A, B^ Values with different letters in the same column indicate significant difference at *p* < 0.05

^a, b^ Values with different letters in the same line indicate significant difference at *p* < 0.05

^1, 2^ Values with different numbers in the same column in the same sample group on the same day indicate significant difference at *p* < 0.05

C: Control candy; KS: Kenger gum-added sugared candy; KSF: Kenger gum-added sugar-free candy; PC: Phycocyanin; 0, 0.1, 0.3, 0.5: Phycocyanin additive percentage

The a_w_ values of soft confectionery products are generally between 0.45–0.60 according to softness or hardness state (Ergun et al. 2010), and in the current study, except for the KS samples, the a_w_ values are within the specified range in other samples. The properties of the components used in the formulation, such as solubility, boiling point, molecular weight, each have an effect on a_w_. Therefore, monitoring the product moisture is critical for producing high-quality soft confectionery with a long shelf life (Cano-Lamadrid et al. 2020; Hartel et al. 2018). In this respect, the use of kenger gum and the new production process did not cause any negative effects on the a_w_ values of the products. It was ensured that the phycocyanin addition could be included in the production in ‘powder’ form, and this prevented the a_w_ values of the products to go beyond the critical limits during the storage period. However, it was determined that the a_w_ values of the samples in KSF group increased due to moisture migration after the 90th day due to the use of sweeteners such as sorbitol and maltitol syrup, which have high hygroscopicity. The results obtained in similar product studies in the literature are consistent with the data in the present research (Da Silva et al. 2016; Thanusan et al. 2018).

### Color analysis results

**Fig. 1:**
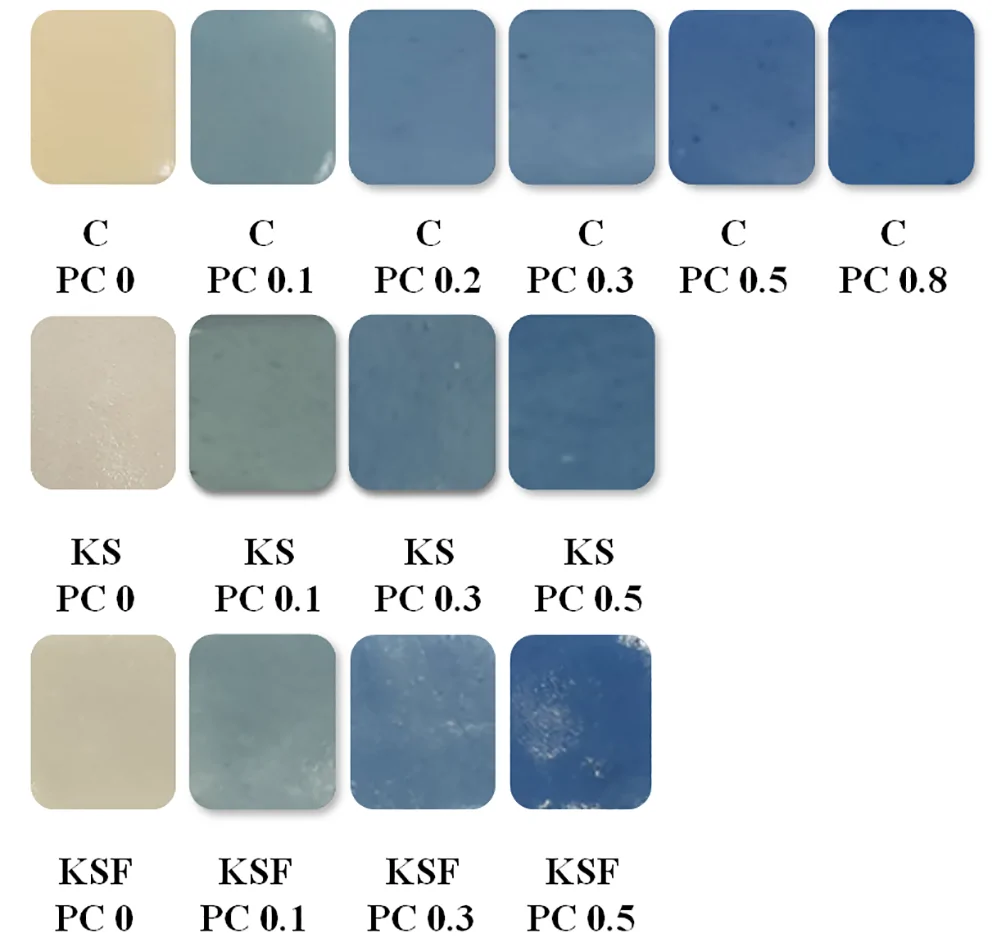
Surface appearance of phycocyanin-free and phycocyanin added soft confectionery products. C: Control, KS: Kenger gum-added sugared toffee type soft candy, KSF: Kenger gum-added sugar-free toffee type soft candy. 0, 0.1, 0.2, 0.3, 0.5, 0.8: Phycocyanin additive percentage. The additives of 0.2% and 0.8% phycocyanin powder were added to the control sample only for trial purposes, and then, the ratios of 0.1%, 0.3% and 0.5% were kept constant in all productions

The surface appearances of all samples are given in Fig. 1, and the L*, a*, b*, and ΔE values are given in Table 2. First, considering the phycocyanin-free samples in all three groups; it was determined that the L* value of the control toffee sample was the highest, followed by KS and KSF samples, respectively (*p* < 0.05). A similar result was also observed in the a* values of the samples (*p* < 0.05). While the control sample had the highest value in terms of + b* value (*p* < 0.05), it was determined that there was no statistical difference between the sugared and sugar-free samples produced with kenger gum (*p* > 0.05). These results proved that the confectionery matrix and its compounds were effective in changing the color parameters (Koohestani et al. 2019). Depending on the storage time, L*, a*, and b* values of phycocyanin-free control and KS samples did not change much, while L* value increased significantly and -a* and + b* values decreased in phycocyanin-free KSF sample. In the literature, differences in perceivable color are analytically classified as very distinct ΔE > 3 (i), distinct 1.5 < ΔE < 3 (ii) and small difference ΔE < 1.5 (iii) (Adekunte et al. 2010). In this context, the ΔE values of the phycocyanin-free control and KS samples were found to be in a small difference, while the difference in the perceivable color of the phycocyanin-free KSF sample was analytically very significant (4.63–10.77) (Table 2). According to these findings, the difference in the color values of phycocyanin-free KS and KSF samples compared to the control toffee was due to the use of kenger gum in the production, and the use of sugar alcohols in the KSF sample had an effect on the color parameters as well.

**Table 2:** Color analysis results of soft candy samples

Sample	L*			a*			b*			ΔE		
	Day(s)			Day(s)			Day(s)			Day(s)		
	1.	90.	150.	1.	90.	150.	1.	90.	150.	1.	90.	150.
**C PC 0**	83.41 ± 0.34^Aa1^	83.40 ± 0.35^Aa1^	83.38 ± 0.36^Aa1^	2.73 ± 0.10^Aa1^	2.71 ± 0.12^Aa1^	2.69 ± 0.08^Aa1^	19.39 ± 0.33^Aa1^	19.41 ± 0.36^Aa1^	19.42 ± 0.29^Aa1^	-	0.03	0.06
**C PC 0.1**	63.13 ± 0.39^Ca2^	61.49 ± 0.49^Cb2^	60.32 ± 0.24^Dc2^	-11.33 ± 0.11^Hc3^	-12.68 ± 0.13^Hb3^	-13.58 ± 0.17^Ga3^	-8.09 ± 0.17^Ea2^	-6.85 ± 0.23^Eb2^	-5.87 ± 0.13^Ec2^	-	2.45	4.22
**C PC 0.3**	53.89 ± 0.66^Ea3^	52.01 ± 0.35^Eb3^	50.83 ± 0.39^FGc3^	-13.15 ± 0.12^Ic4^	-14.25 ± 0.20^Ib4^	-14.88 ± 0.28^Ha4^	-18.55 ± 0.22^Ha3^	-16.91 ± 0.12^Hbb3^	-16.07 ± 0.24^Hc3^	-	2.72	4.30
**C PC 0.5**	48.64 ± 0.41^Ga4^	46.71 ± 0.58^Gb4^	45.57 ± 0.33^Hc4^	-10.43 ± 0.09^Gc2^	-11.19 ± 0.25^Gb2^	-11.75 ± 0.20^EFa2^	-23.18 ± 0.13^Ia4^	-21.37 ± 0.21^Jb4^	-20.33 ± 0.26^Jc4^	-	2.76	4.40
**KS PC 0**	68.19 ± 0.42^Ba1^	68.25 ± 0.43^Ba1^	67.86 ± 0.37^Ca1^	-0.28 ± 0.08^Ba1^	-0.30 ± 0.05^Ba1^	-0.39 ± 0.06^Ba1^	9.83 ± 0.19^Ba1^	10.26 ± 0.29^Ba1^	10.32 ± 0.36^Ba1^	-	0.43	0.60
**KS PC 0.1**	58.31 ± 0.36^Da2^	57.51 ± 0.25^Db2^	57.04 ± 0.28^Eb2^	-7.93 ± 0.20^Db2^	-8.87 ± 0.21^Da2^	-9.30 ± 0.14^Ca2^	-0.74 ± 0.10^Cc2^	0.11 ± 0.25^Db2^	0.67 ± 0.07^Da2^	-	1.46	2.34
**KS PC 0.3**	51.35 ± 0.13^Fa3^	50.29 ± 0.21^Fb3^	49.66 ± 0.14^Gc3^	-10.76 ± 0.11^Gc3^	-11.61 ± 0.18^Gb3^	-12.11 ± 0.23^Fa3^	-9.27 ± 0.45^Fa3^	-8.26 ± 0.24^Fb3^	-7.68 ± 0.26^Fb3^	-	1.69	2.68
**KS PC 0.5**	45.94 ± 0.18^Ha4^	44.69 ± 0.23^Hb4^	44.08 ± 0.15^Ic4^	-10.61 ± 0.14^Gb3^	-11.55 ± 0.13^Ga3^	-11.89 ± 0.10^Fa3^	-14.22 ± 0.10^Ga4^	-13.20 ± 0.18^Gb4^	-12.74 ± 0.25^Gb4^	-	1.87	2.71
**KSF PC 0**	63.47 ± 0.52^Cc1^	67.89 ± 0.34^Bb1^	73.57 ± 1.23^Ba1^	-1.83 ± 0.05^Ca1^	-1.51 ± 0.08^Cb1^	-0.93 ± 0.03^Bc1^	9.17 ± 0.21^Ba1^	7.85 ± 0.50^Cb1^	5.52 ± 0.18^Cc1^	-	4.63	10.77
**KSF PC 0.1**	64.37 ± 0.56^Ca1^	61.57 ± 0.37^Cb2^	60.45 ± 0.36^Dc2^	-8.36 ± 0.17^Ec2^	-10.41 ± 0.23^Fb3^	-11.29 ± 0.31^Ea3^	-4.65 ± 0.18^Dc2^	-6.93 ± 0.28^Eb2^	-7.80 ± 0.33^Fa2^	-	4.15	5.82
**KSF PC 0.3**	55.03 ± 0.33^Ea2^	52.45 ± 0.22^Eb3^	51.28 ± 0.32^Fc3^	-9.50 ± 0.19^Fc3^	-11.12 ± 0.38^Gb4^	-12.00 ± 0.23^Fa4^	-14.54 ± 0.13^Gc3^	-16.74 ± 0.36^Hb3^	-17.76 ± 0.38^Ia3^	-	3.75	5.53
**KSF PC 0.5**	49.95 ± 0.71^Ga3^	46.97 ± 0.37^Gb4^	45.61 ± 0.33^Hc4^	-8.08 ± 0.16^DEc2^	-9.67 ± 0.11^Eb2^	-10.70 ± 0.20^Da2^	-18.42 ± 0.30^Hb4^	-20.32 ± 0.46^Ia4^	-21.24 ± 0.43^Ka4^	-	3.88	5.80

Data represent average values ± standard deviation of three independent samples

^A, B^ Values with different letters in the same column indicate significant difference at *p* < 0.05

^a, b^ Values with different letters in the same line indicate significant difference at *p* < 0.05

^1, 2^ Values with different numbers in the same column within the same sample group on the same day indicate significant difference at *p* < 0.05

C: Control candy; KS: Kenger gum-added sugared candy; KSF: Kenger gum-added sugar-free candy; PC: Phycocyanin; 0, 0.1, 0.3, 0.5: Phycocyanin additive percentage

Considering the color analysis results of the samples colored with 0.1, 0.3, and 0.5 g/100 g of powder phycocyanin, it was observed that as the amount of phycocyanin increased, the L* values in the samples included in all groups decreased significantly (*p* < 0.05). While there was no statistical difference between the L* values of the control and KSF samples with the same phycocyanin content, the values were lower in the samples belonging to the KS group and this situation did not change during storage. However, it was determined that the L* values of the samples decreased depending on the storage time in all groups (*p* < 0.05). On the other hand, the increase in the addition of phycocyanin increased the a* values of all samples in the negative direction (greenness), and at 0.5 g/100 g concentration, contrary to the expectations, the -a* values of the samples decreased compared to the 0.3 g/100 g concentration. In addition, it was determined that the -a* values of the colored samples in all groups increased depending on the storage time (*p* < 0.05).

In the study, the natural blue color provided by the phycocyanin addition to the samples can be evaluated with the -b* (blueness) value. As seen in Table 2, it was determined that -b* values increased in parallel with the increase in the amount of phycocyanin additive in colored samples in all groups (*p* < 0.05). According to the results, it was determined that the -b* values on the first day decreased in the control and KS samples towards the end of the storage period, while it increased in contrast to the trend observed in the KSF samples, which was assumed to be caused by the different sweetening agents in the formulation (Table 2). In the meantime, the ΔE values of the samples belonging to all three groups increased with the storage time and the greatest change was observed in the KSF samples.

There is great demand to limit the use of artificial colorants in confectionery products or switch to natural ones in conjunction with the shift in consumer preferences and expectations. In this regard, it is stated that stable microalgae-based colorants have an important commercialization potential in the confectionery industry (Konar et al. 2022). Considering the results obtained in the present study, it was determined that blue colored phycocyanin, which is a protein structure compound of microalgae origin, can be used in conventional toffee type soft candies if the temperature is controlled by adding it during the pulling step, and thus, it can preserve its natural blue color for a long time during storage. However, from a sectoral point of view, homogeneous dispersibility of the phycocyanin powder should be investigated due to the high viscosity of candy mass in the pulling step. On the other hand, in the new production technique discovered using kenger gum, phycocyanin can be successfully used in the process at low temperatures thanks to the z-blade mixer without any negative effects on its coloring capacity and homogeneity concerns.

### Texture analysis results

The texture profile analysis (TPA) results of the samples are given in Table 3. The production of soft confectionery with optimum hardness value is a critical factor in terms of consumer appeal and sales strategy. In this regard, considering the hardness results, the values ​​of the phycocyanin-free control, KS, and KSF samples were determined as 15.039 N, 5.068 N, and 18.387 N, respectively (*p* < 0.05). In a study, Da Silva et al. (2016) emphasized that chewy candy samples containing isomalt and maltitol combination had high hardness, which adversely affected the chewiness and product texture, despite having appropriate water activity values. Similarly, in the present study, the highest hardness values were obtained in the samples belonging to the KSF group since the formulation consisted entirely of polyols. However, it was observed that the addition of phycocyanin did not cause a difference in the hardness values of the samples in the same group (*p* > 0.05). In addition, it was determined that there was a linear increase in the hardness values ​​of all samples depending on the storage time (*p* < 0.05), which coincides with previous studies showing that moisture content plays a pivotal role in determining the hardness of texture (Delgado and Bañón 2015; Kaur et al. 2023).

Chewiness is the another parameter affecting the product appeal in soft candies. Similar to the hardness results, the highest chewiness values ​​were also in KSF samples, followed by the samples in the control and KS groups (*p* < 0.05). It was determined that the use of phycocyanin did not cause any difference in the chewiness values ​​of the samples within the groups (*p* > 0.05), however the values increased depending on the storage period and the highest results were recorded on the 150th day (*p* < 0.05) (Table 3), indicating that more energy would be required to chew the samples (Aggarwal et al. 2022). The cohesiveness values varied between samples due to differences in the product formulation (*p* < 0.05). During the storage period, the cohesiveness values of the samples decreased in all groups and KSF samples had the highest values throughout the entire period (*p* < 0.05). Based on the findings from different products in the literature, some studies indicate that there is a negative (Clarke and Farrell 2000), positive up to a critical point (Tobin et al. 2017), or completely proportional (Zheng et al. 2016) relationship between the product’s cohesiveness and moisture content. From this point of view, considering the current results, it was seen that the cohesiveness values of the samples in all groups decreased in response to the decrease in water activity values, which was consistent with the results of Kaur et al. (2023). As the last parameter, the highest adhesiveness values were found in the KSF group (*p* < 0.05) on the first day, while the samples in the control and KS groups were detected to be similar (*p* > 0.05). Although there were differences in the adhesiveness value of the samples depending on the storage, the same trend was observed at the end of the period and the highest values were determined on the 150th day in the samples in the KSF group (*p* < 0.05). In addition to all these, there was no statistical finding that the amount of phycocyanin addition had an effect on the cohesiveness and adhesiveness values of the samples.

**Table 3:** Texture analysis results of soft candy samples

Sample	Hardness (*N*)			Chewiness (*N*)			Cohesiveness			Adhesiveness (*N*.sec)		
	Day(s)			Day(s)			Day(s)			Day(s)		
	1.	90.	150.	1.	90.	150.	1.	90.	150.	1.	90.	150.
**C PC 0**	15.039 ± 0.246^Bc1^	35.596 ± 0.854^Bb1^	76.948 ± 0.270^Ba1^	1.514 ± 0.172^Bc1^	3.666 ± 0.153^Bb1^	6.171 ± 0.497^Ba1^	0.163 ± 0.004^Ca1^	0.133 ± 0.005^Bb1^	0.106 ± 0.006^Cc1^	-0.106 ± 0.006^Ba1^	-0.033 ± 0.006^Bb1^	-0.018 ± 0.002^Bc1^
**C PC 0.1**	15.130 ± 0.184^Bc1^	35.778 ± 0.812^Bb1^	77.017 ± 0.304^Ba1^	1.539 ± 0.057^Bc1^	3.619 ± 0.145^Bb1^	6.392 ± 0.153^Ba1^	0.166 ± 0.001^Ca1^	0.134 ± 0.005^Bb1^	0.109 ± 0.001^Cc1^	-0.100 ± 0.002^Ba1^	-0.032 ± 0.004^Bb1^	-0.017 ± 0.003^Bc1^
**C PC 0.3**	15.281 ± 0.487^Bc1^	35.816 ± 0.787^Bb1^	77.104 ± 0.299^Ba1^	1.566 ± 0.144^Bc1^	3.735 ± 0.101^Bb1^	6.416 ± 0.551^Ba1^	0.168 ± 0.005^Ca1^	0.139 ± 0.005^Bb1^	0.110 ± 0.005^Cc1^	-0.103 ± 0.017^Ba1^	-0.031 ± 0.002^Bb1^	-0.016 ± 0.001^Bc1^
**C PC 0.5**	15.064 ± 0.332^Bc1^	35.927 ± 0.841^Bb1^	76.976 ± 0.358^Ba1^	1.476 ± 0.098^Bc1^	3.786 ± 0.033^Bb1^	6.216 ± 0.039^Ba1^	0.165 ± 0.006^Ca1^	0.136 ± 0.005^Bb1^	0.108 ± 0.004^Cc1^	-0.104 ± 0.010^Ba1^	-0.031 ± 0.002^Bb1^	-0.017 ± 0.001^Bc1^
**KS PC 0**	5.068 ± 0.169^Cc1^	20.245 ± 0.413^Cb1^	32.625 ± 0.640^Ca1^	0.742 ± 0.052^Cc1^	2.379 ± 0.090^Bb1^	3.051 ± 0.180^Ca1^	0.230 ± 0.007^Ba1^	0.161 ± 0.007^Bb1^	0.138 ± 0.004^Bc1^	-0.103 ± 0.001^Ba1^	-0.043 ± 0.003^Bb1^	-0.021 ± 0.002^Bc1^
**KS PC 0.1**	5.159 ± 0.345^Cc1^	20.491 ± 0.478^Cb1^	32.717 ± 0.679^Ca1^	0.773 ± 0.063^Cc1^	2.556 ± 0.289^Bb1^	3.158 ± 0.145^Ca1^	0.237 ± 0.005^Ba1^	0.169 ± 0.012^Bb1^	0.145 ± 0.008^Bc1^	-0.100 ± 0.004^Ba1^	-0.040 ± 0.002^Bb1^	-0.019 ± 0.002^Bc1^
**KS PC 0.3**	5.249 ± 0.333^Cc1^	20.356 ± 0.467^Cb1^	32.652 ± 0.675^Ca1^	0.856 ± 0.149^Cc1^	2.438 ± 0.615^Bb1^	3.017 ± 0.127^Ca1^	0.252 ± 0.027^Ba1^	0.164 ± 0.034^Bb1^	0.140 ± 0.004^Bb1^	-0.097 ± 0.005^Ba1^	-0.042 ± 0.002^Bb1^	-0.022 ± 0.001^Bc1^
**KS PC 0.5**	5.115 ± 0.234^Cc1^	20.415 ± 0.646^Cb1^	32.569 ± 0.884^Ca1^	0.793 ± 0.049^Cc1^	2.495 ± 0.332^Bb1^	2.947 ± 0.013^Ca1^	0.246 0.004 ± ^Ba1^	0.165 ± 0.013^Bb1^	0.136 ± 0.004^Bc1^	-0.099 ± 0.006^Ba1^	-0.043 ± 0.005^Bb1^	-0.020 ± 0.001^Bc1^
**KSF PC 0**	18.387 ± 0.109^Ac1^	134.883 ± 1.042^Aa1^	115.264 ± 0.657^Ab1^	9.509 ± 0.329^Ac1^	55.249 ± 1.406^Aa1^	40.936 ± 0.670^Ab1^	0.515 ± 0.011^Aa1^	0.413 ± 0.010^Ab1^	0.358 ± 0.004^Ac1^	-5.275 ± 0.149^Aa1^	-4.064 ± 0.204^Ab1^	-4.310 ± 0.059^Ab1^
**KSF PC 0.1**	18.463 ± 0.182^Ac1^	134.951 ± 0.794^Aa1^	115.354 ± 0.791^Ab1^	9.683 ± 0.123^Ac1^	55.976 ± 2.338^Aa1^	41.464 ± 0.911^Ab1^	0.518 ± 0.007^Aa1^	0.420 ± 0.018^Ab1^	0.362 ± 0.005^Ac1^	-5.179 ± 0.175^Aa1^	-3.941 ± 0.228^Ab1^	-4.295 ± 0.034^Ab1^
**KSF PC 0.3**	18.363 ± 0.085^Ac1^	134.916 ± 0.426^Aa1^	115.419 ± 0.968^Ab1^	9.521 ± 0.137^Ac1^	55.567 ± 2.031^Aa1^	41.238 ± 0.899^Ab1^	0.512 ± 0.004^Aa1^	0.416 ± 0.043^Ab1^	0.360 ± 0.005^Ab1^	-5.321 ± 0.217^Aa1^	-4.039 ± 0.159^Ab1^	-4.267 ± 0.081^Ab1^
**KSF PC 0.5**	18.412 ± 0.133^Ac1^	135.105 ± 0.630^Aa1^	115.478 ± 0.551^Ab1^	9.576 ± 0.280^Ac1^	55.850 ± 2.039^Aa1^	41.611 ± 0.393^Ab1^	0.517 ± 0.011^Aa1^	0.419 ± 0.013^Ab1^	0.365 ± 0.003^Ac1^	-5.207 ± 0.155^Aa1^	-3.979 ± 0.141^Ab1^	-4.243 ± 0.045^Ab1^

Data represent average values ± standard deviation of three independent samples

^A, B^ Values with different letters in the same column indicate significant difference at *p* < 0.05

^a, b^ Values with different letters in the same line indicate significant difference at *p* < 0.05

^1, 2^ Values with different numbers in the same column within the same sample group on the same day indicate significant difference at *p* < 0.05

C: Control candy; KS: Kenger gum-added sugared candy; KSF: Kenger gum-added sugar-free candy; PC: Phycocyanin; 0, 0.1, 0.3, 0.5: Phycocyanin additive percentage

### Sensory analysis results

**Fig. 2:**
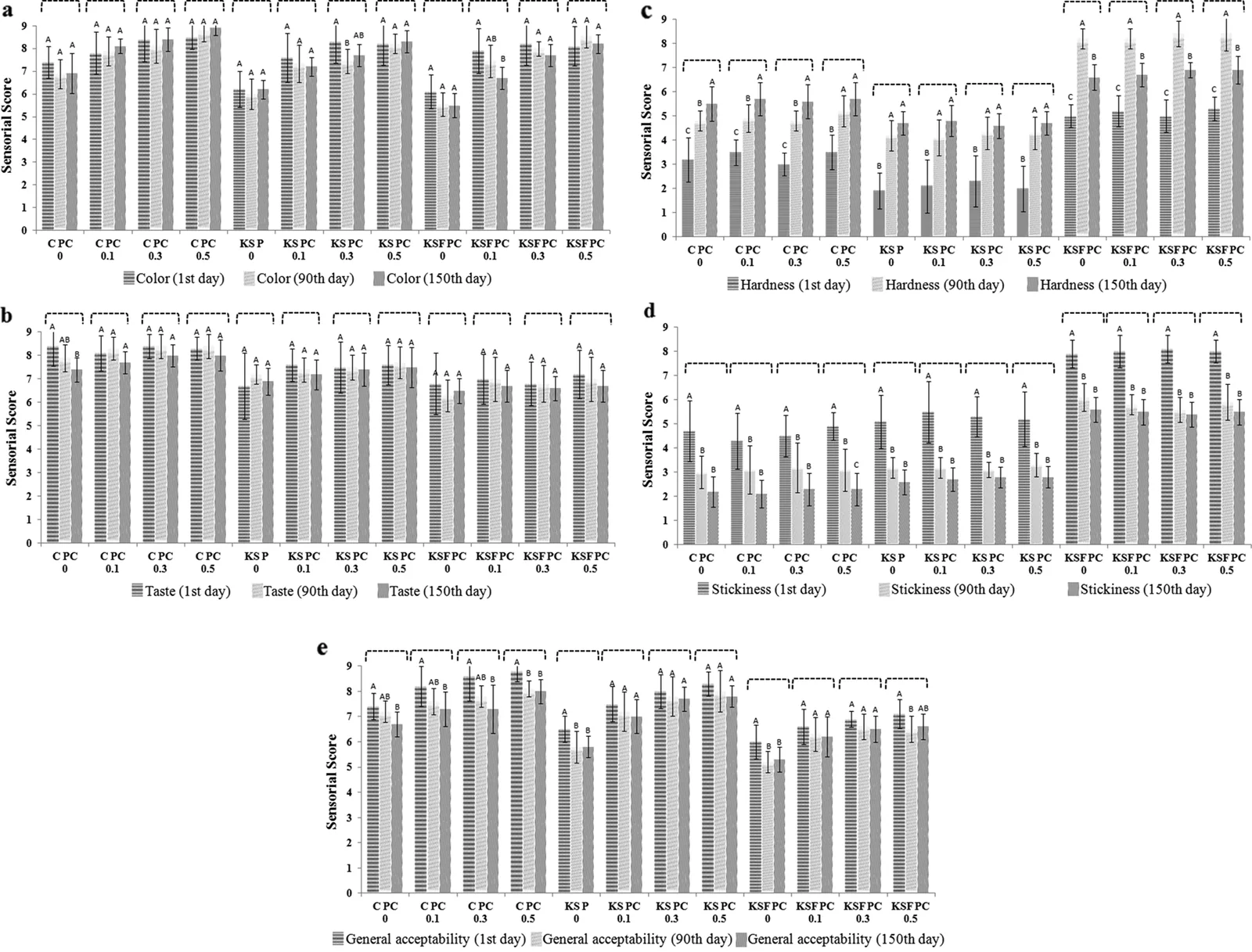
Sensory analysis results of candy samples. C: Control candy; KS: Kenger gum-added sugared candy; KSF: Kenger gum-added sugar-free candy; PC: Phycocyanin; 0, 0.1, 0.3, 0.5: Phycocyanin additive percentage. The bars represent the average values ± standard deviation of the relevant sensory scores. Different capital letters above the bars indicate statistical significance between different storage times of the same sample (*p* < 0.05)

Sensory analysis results of all samples during the 5-month storage period are given in Fig. 2. As shown in Fig. 2a, it was determined that the color scores of phycocyanin-free control (PC0), KS (PC0), and KSF (PC0) samples changed between 7.4–6.9, 6.0–6.2 and 6.1–5.5, respectively, during the storage period. Meanwhile, there was a slight decrease in scores over time, but no significant difference was observed in the scores between the samples (*p* > 0.05). The unique color of the kenger gum and the difference in the composition of these novel samples compared to the control sample may have influenced the color scores of the panelists. On the other hand, the use of phycocyanin as a natural blue colorant was appreciated by the panelists in all three groups, and as the amount of phycocyanin additive increased in the formulation, the color liking of the samples increased compared to the phycocyanin-free samples (*p* < 0.05). It should be noted that the color scores of all phycocyanin-added samples were in the high score category (6.7–8.9) throughout the study. In addition, during the entire storage period, there was no significant change in the scores of all colored samples belonging to the all groups (*p* > 0.05), except for a slight decrease in the scores of KS (PC0.3) and KSF (PC0.1) samples (*p* < 0.05) (Fig. 2a).

Considering the taste scores (Fig. 2b), phycocyanin-free KS and KSF samples were statistically similar in taste but slightly lower than the control sample. It can be stated that this situation is caused by kenger gum and squalene, which have a unique flavor, found in the composition of these two samples. Apart, it was determined that there was no change in the taste of all samples belonging to the groups, except sample C (PC0), during the storage period (*p* < 0.05). It is known that modifications to the formulation and production process have a significant effect on the final product’s physical, textural, sensory, and aroma release qualities (Konar et al. 2022).

According to the sensory hardness (Fig. 2c), it was determined that the samples in the KSF group (5.0-8.4) had the highest scores during storage, followed by the samples in the control (3.0-5.7) and KS (1.9–4.8) groups, respectively. This situation is caused by the significant effect of sugar alcohols in the formulation of KSF samples on the product texture. Depending on the storage period, there was a gradual increase in the hardness scores towards the end of the period in all samples belonging to the control and KS groups, while in the samples from the KSF group, there was a decrease in sensory hardness scores on the 150th day after the increase on the 90th day, but it was observed that the scores in these samples were higher at the end of the storage period than on the first day (Fig. 2c) (*p* < 0.05). These results were closely related to the a_w_ results of the samples and were consistent with the data obtained in Table 1. In other words, the decrease in a_w_ values ​​of the samples belonging to the KSF group until the 90th day and the increase on the 150th day had an effect on the sensory hardness results. In the meantime, the amount of phycocyanin did not alter the sensory hardness scores of the samples within the groups.

The sensory stickiness scores of the samples in the control (2.1–4.9) and KS (2.6–5.5) groups were found to be similar and the highest scores were given to the samples belonging to the KSF (5.4–8.1) group (Fig. 2d). According to the storage time, the scores of all samples decreased and this was associated with moisture loss of the samples. Although these results are seen as an advantage, the use of additives that stabilize the moisture balance of kenger gum-added samples may be essential in future studies. On the other hand, it can be stated that the increase in phycocyanin concentration did not change the sensory stickiness scores of the samples within the groups.

Considering the general acceptability scores, first, the phycocyanin-free KS (5.8–6.5) and KSF (5.2-6.0) samples were found to be relatively lower than the control sample (6.7–7.4) (Fig. 2e). It was concluded that the differences in composition such as kenger gum, squalene and sugar alcohols, which were effective factors in other sensorial parameters, as well as the instrumental hardness and adhesiveness values ​​of these novel samples, affected the perception of the panelists. On the other hand, as the amount of phycocyanin usage increased in all groups, it was determined that the samples with intensifying blue color density were better appreciated by panelists and the general acceptability scores increased. In contrast, there was a decrease in the general acceptability scores of all samples except for the colored samples of the KS group and KSF PC0.3 and KSF PC0.5 samples during the storage period (*p* < 0.05). Kaur et al. (2023) found that all sensory parameters evaluated in toffee samples decreased consistently as storage time increased. However, considering that this decrease remained above 5 points on the hedonic scale, it can be stated that the use of both kenger gum and phycocyanin in toffee type soft candy production provides high consumption appeal.

## Conclusion

In the present study, kenger gum was utilized in the production of toffee type soft candy and phycocyanin powder was added to the samples as a natural blue color source. Within the scope, various quality analyses were carried out on the samples during the 5-month storage period at 20 ± 2 °C. According to the results, it was determined that the differences in the water activity of the samples were due to the component differences in the formulation rather than the use of kenger gum. Considering the color analysis results, adding phycocyanin at low temperature (40 °C) to the samples using a z-blade mixer provided a great advantage in terms of pigment stability during storage. In the texture analysis, KSF samples were recorded as the hardest samples due to the presence of sugar alcohols in the production. The use of both kenger gum and phycocyanin in soft candies was found to be acceptable by the panelists. However, using various additives such as preservatives, humectants, antioxidants, fats, and emulsifiers to increase the product’s appeal in toffee type soft candy samples with kenger gum should be investigated, and future studies should focus on the functional characteristics of phycocyanin-added confectionery products. As a result, it can be stated that kenger gum was effectively employed in the manufacturing of toffee type soft confectionery, and a novel product was introduced to the sector.

## Data Availability

The data that support the findings of this study are available from the corresponding author upon reasonable request.

## Abbreviations

FDA: U.S. Food and Drug Administration
KS: Kenger gum-added sugared soft candy
KSF: Kenger gum-added sugar-free soft candy
TPA: Texture profile analysis
UK: United Kingdom
USA: United States of America
